# Supplementary material for: Eleutheroside E alleviates cisplatin-induced ototoxicity by down-regulating MAPK/NF-κB/NLRP3 signaling pathway and inhibiting cochlear cell pyroptosis
Source: Commun Biol. 2026 Jan 8;9:214. doi: 10.1038/s42003-025-09490-x (PMC12894672; doi:10.1038/s42003-025-09490-x)
Supplement: Supplementary file 2 — Supplementary information [file 42003_2025_9490_MOESM2_ESM.pdf]

Supplementary Information

Eleutheroside E alleviates Cisplatin-Induced ototoxicity by down-regulating MAPK/NF-κB/NLRP3 signaling pathway and inhibiting cochlear cell pyroptosis

Ya'nan Zhang, Ling Lu, Busheng Tong, Jingjing Wang, Kunjian Liu, Jialiang Zhang, Di Zhang, Meihui Tian, Weifang Sun, Huan Liu, Ping Wang, Maoli Duan, Yong Tang

This PDF file includes:

Supplementary Figure 1

Supplementary Table 1

Full unedited gels

Supplementary Figure 1 Flowchart of the overall research strategy

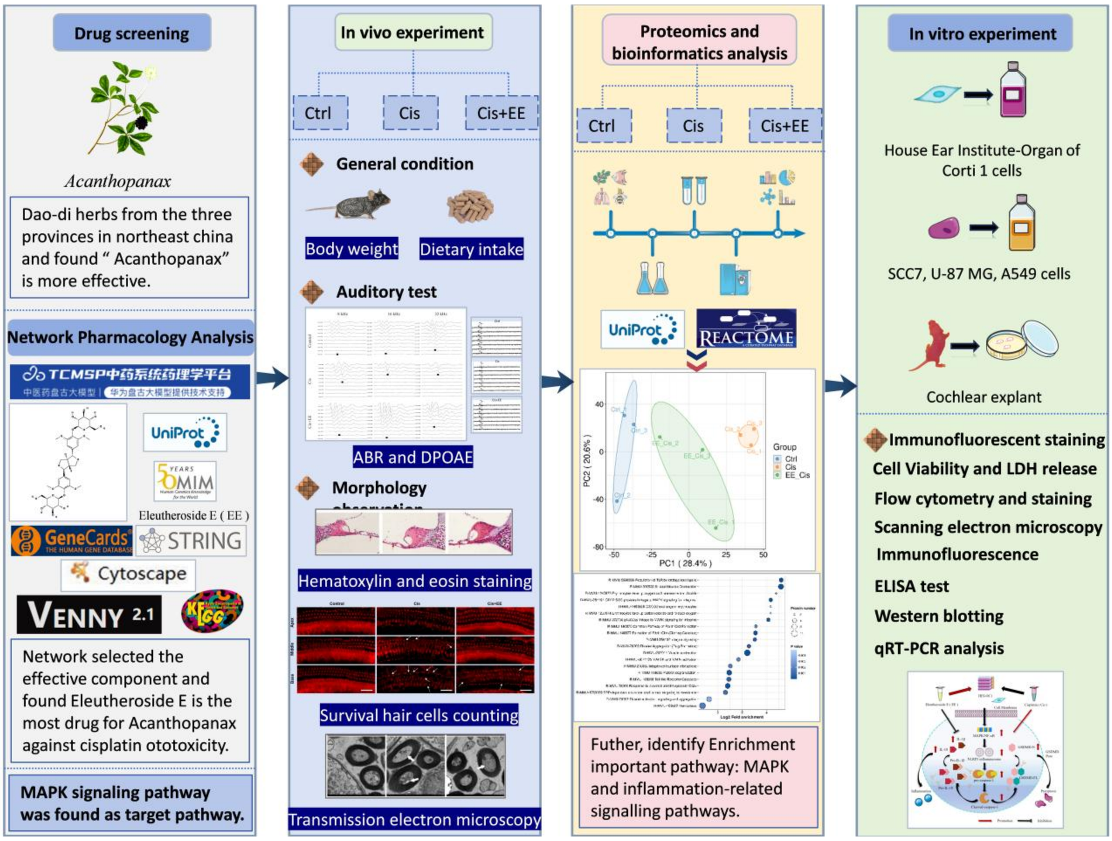

Supplementary Table 1 The primer sequences for *Caspase1,ASC,NLRP3,IL-1β,IL-18,GSDMD and β-actin*.

**Item**	**Primer（5' to 3'）**	**Length (bp)**
IL-18-F	CTGAATCCTGCCCCAGTGC	174
IL-18-R	CGGGGCCTGAGGATTATAGC	
IL-1β-F	GCCACCTTTTGACAGTGATGAG	95
IL-1β-R	GACAGCCCAGGTCAAAGGTT	
NLRP3-F	TTTGTACCCAAGGCTGCTATCT	137
NLRP3-R	CACTCGTCATCTTCAGCAGCA	
ASC-F	GTCTTAGGGGCGGAAACCAA	178
ASC-R	CCGCGGTCACCTTTTACTCT	
Caspace1-F	CCTGTCAGGGGCTCACTTTT	74
Caspace1-R	TCCAAGTCACAAGACCAGGC	
GSDMD-F	GATCAAGGAGGTAAGCGGCA	195
GSDMD-R	CACTCCGGTTCTGGTTCTGG	
β-actin-F	TGAGCTGCGTTTTACACCCT	198
β-actin-R	GCCTTCACCGTTCCAGTTTT	

Full unedited gel For Figure 8a

P-ERK

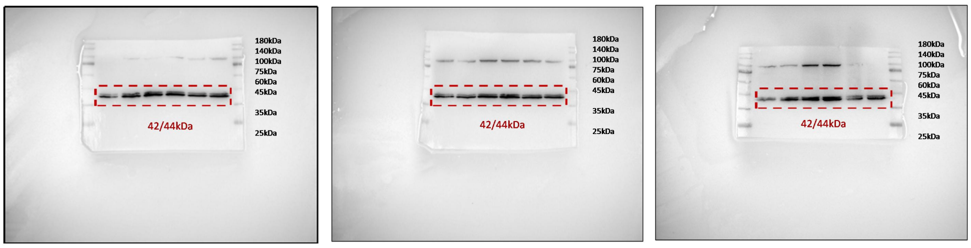

ERK

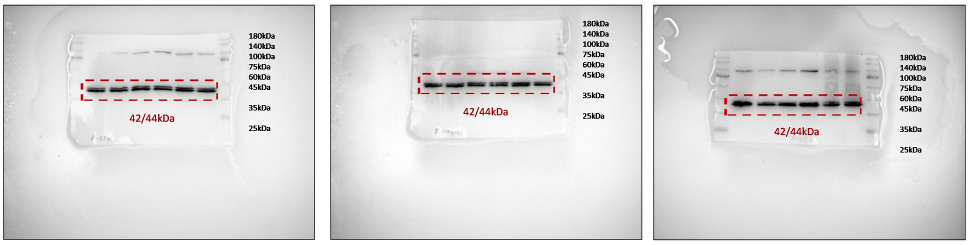

P-P38

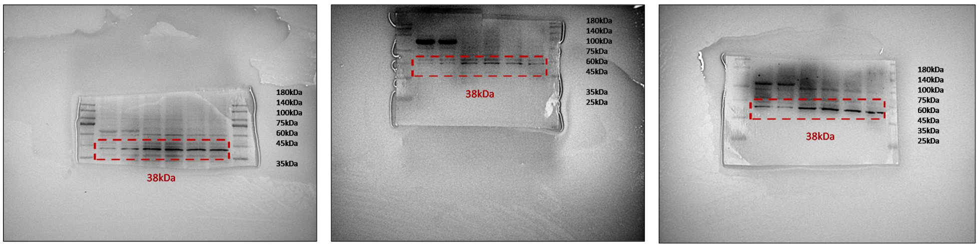

P38

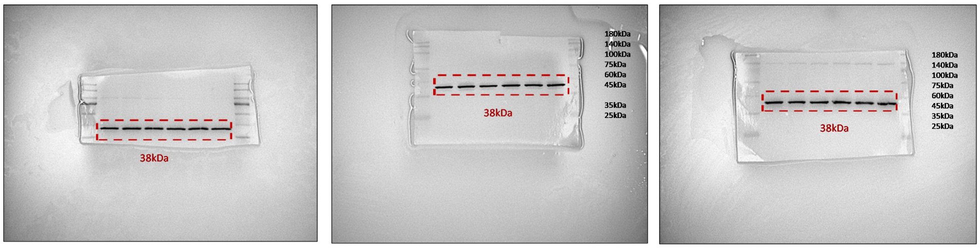

P-JNK

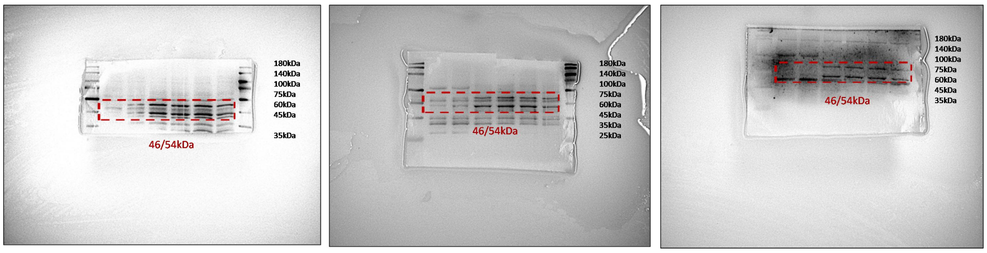

JNK

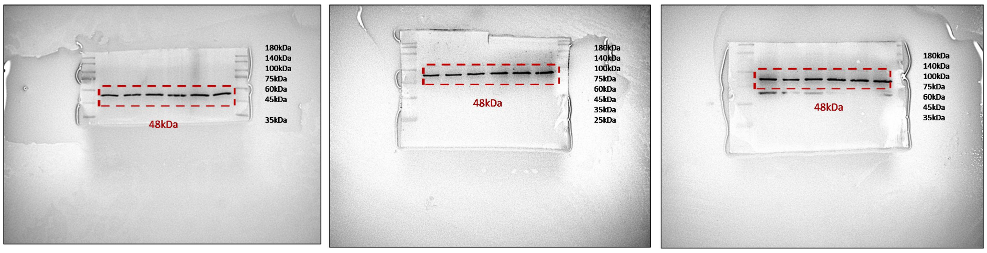

Full unedited gel For Figure 8e

IκBα

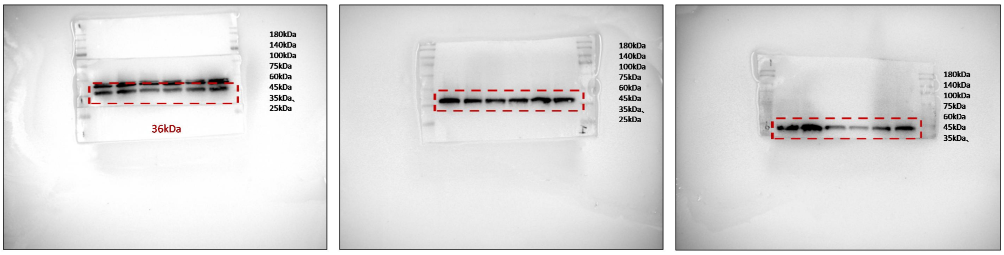

β-actin

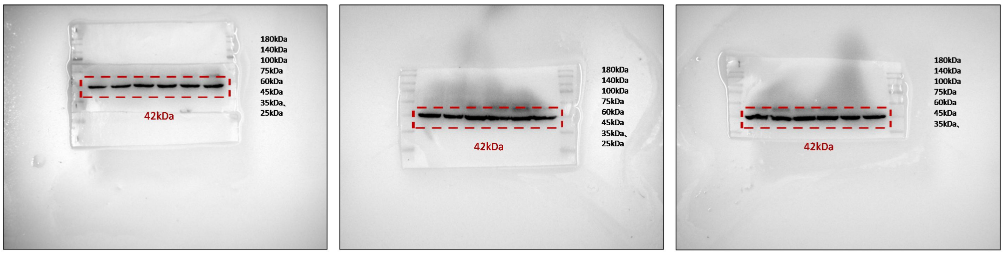

NF-κB-P65

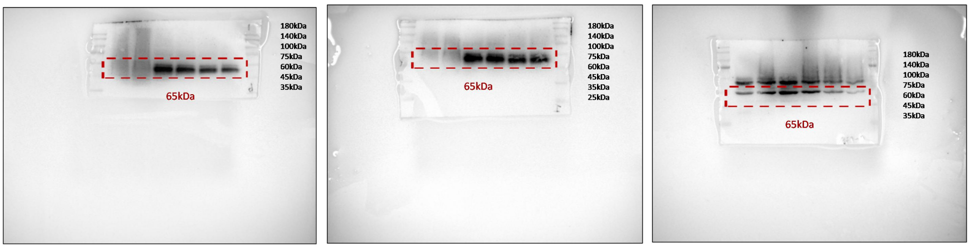

β-actin

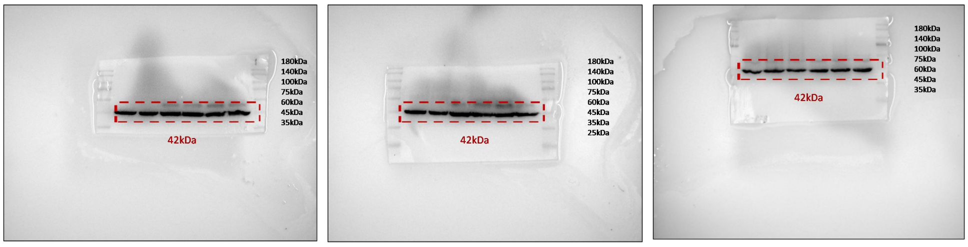

Full unedited gel For Figure 8h

NLRP3

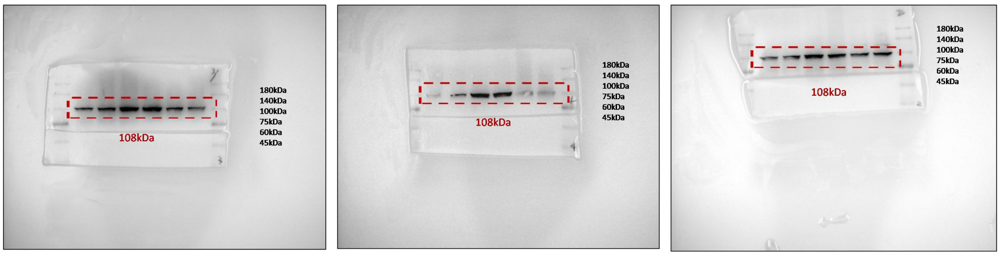

β-actin

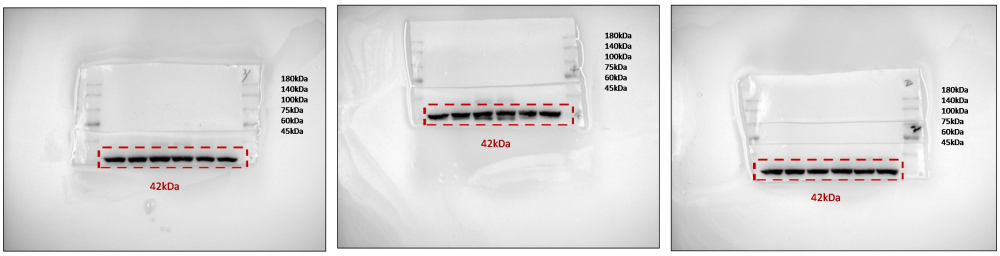

ASC

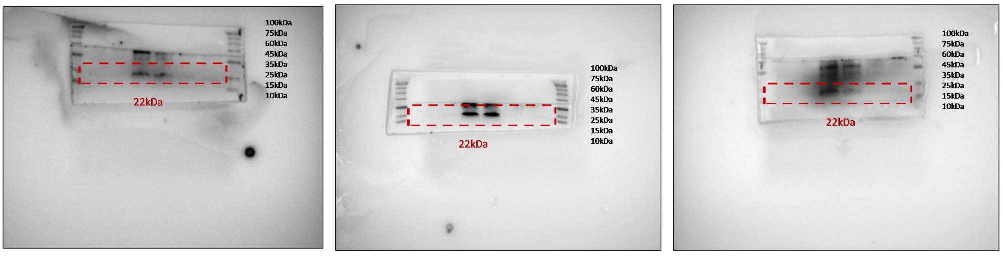

β-actin

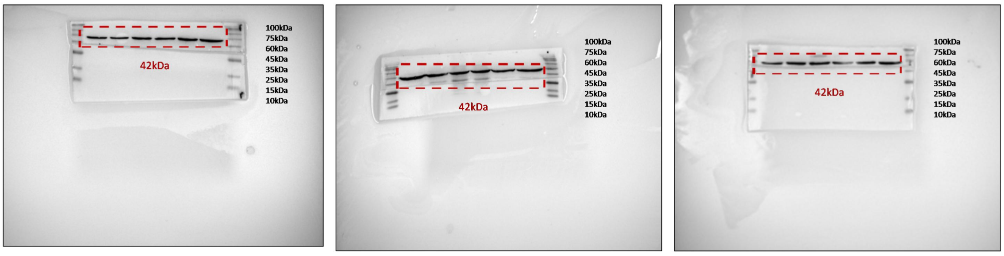

Cleaved-caspase-1

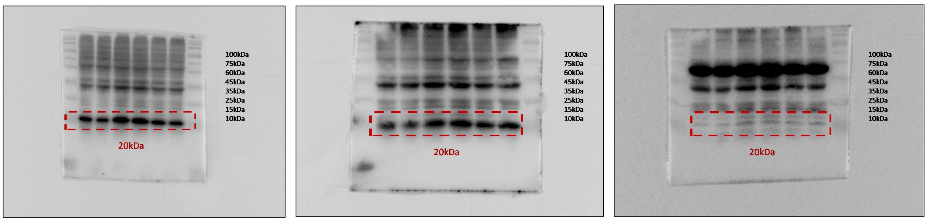

Pro-caspase-1

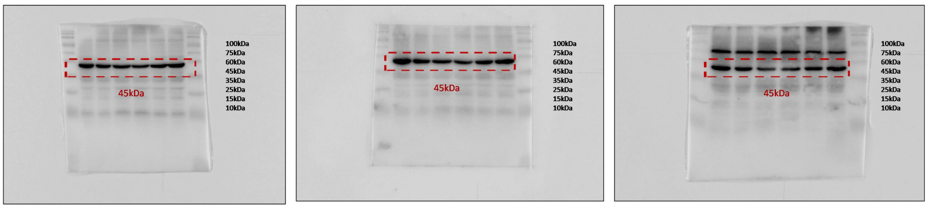

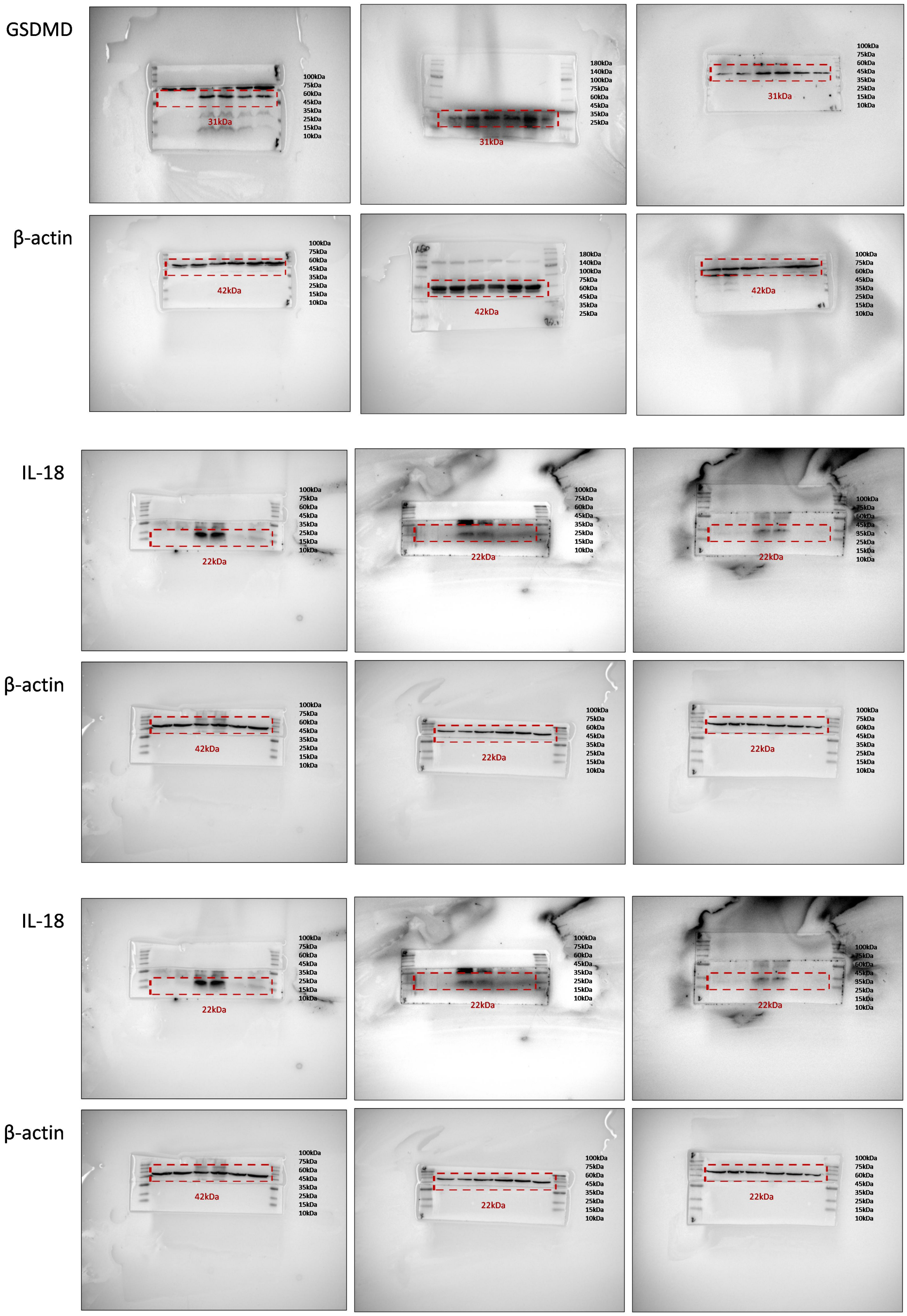
GSDMD
31kDa
31kDa
31kDa
β-actin
42kDa
42kDa
42kDa
IL-18
22kDa
22kDa
22kDa
β-actin
42kDa
22kDa
22kDa
IL-18
22kDa
22kDa
22kDa
β-actin
42kDa
22kDa
22kDa
