# Supplementary material for: Eleutheroside E alleviates cisplatin-induced ototoxicity by down-regulating MAPK/NF-κB/NLRP3 signaling pathway and inhibiting cochlear cell pyroptosis
Source: Commun Biol. 2026 Jan 8;9:214. doi: 10.1038/s42003-025-09490-x (PMC12894672; doi:10.1038/s42003-025-09490-x)
Supplement: Supplementary file 3 — Description of Additional Supplementary Files [file 42003_2025_9490_MOESM3_ESM.pdf]

Description of Additional Supplementary Files:

File: Supplementary Data 1

Description: This file (Supplementary Data 1) contains the source data and statistical analysis underlying the figures presented in the manuscript.
