# Supplementary material for: Eleutheroside E alleviates cisplatin-induced ototoxicity by down-regulating MAPK/NF-κB/NLRP3 signaling pathway and inhibiting cochlear cell pyroptosis
Source: Commun Biol. 2026 Jan 8;9:214. doi: 10.1038/s42003-025-09490-x (PMC12894672; doi:10.1038/s42003-025-09490-x)
Supplement: Supplementary file 5 — nr-reporting-summary [file 42003_2025_9490_MOESM5_ESM.pdf]

nature portfolio

Corresponding author(s): Ya'nan Zhang, Ling Lu, Busheng Tong, Jingjing Wang, Kunjian Liu, Jialiang Zhang, Di Zhang, Meihui Tian, Weifang Sun, Huan Liu, Ping Wang*, Maoli Duan*, Yong Tang*

Last updated by author(s): Nov 27, 2025

Reporting Summary

Nature Portfolio wishes to improve the reproducibility of the work that we publish. This form provides structure for consistency and transparency in reporting. For further information on Nature Portfolio policies, see our Editorial Policies and the Editorial Policy Checklist.

Statistics

For all statistical analyses, confirm that the following items are present in the figure legend, table legend, main text, or Methods section.

n/a	Confirmed	
☐	☒	The exact sample size (*n*) for each experimental group/condition, given as a discrete number and unit of measurement
☐	☒	A statement on whether measurements were taken from distinct samples or whether the same sample was measured repeatedly
☐	☒	The statistical test(s) used AND whether they are one- or two-sided<br>*Only common tests should be described solely by name; describe more complex techniques in the Methods section.*
☒	☐	A description of all covariates tested
☐	☒	A description of any assumptions or corrections, such as tests of normality and adjustment for multiple comparisons
☐	☒	A full description of the statistical parameters including central tendency (e.g. means) or other basic estimates (e.g. regression coefficient) AND variation (e.g. standard deviation) or associated estimates of uncertainty (e.g. confidence intervals)
☐	☒	For null hypothesis testing, the test statistic (e.g. *F*, *t*, *r*) with confidence intervals, effect sizes, degrees of freedom and *P* value noted<br>*Give P values as exact values whenever suitable.*
☒	☐	For Bayesian analysis, information on the choice of priors and Markov chain Monte Carlo settings
☒	☐	For hierarchical and complex designs, identification of the appropriate level for tests and full reporting of outcomes
☒	☐	Estimates of effect sizes (e.g. Cohen's *d*, Pearson's *r*), indicating how they were calculated

Our web collection on statistics for biologists contains articles on many of the points above.

Software and code

Policy information about availability of computer code

Data collection	N/A
Data analysis	N/A

For manuscripts utilizing custom algorithms or software that are central to the research but not yet described in published literature, software must be made available to editors and reviewers. We strongly encourage code deposition in a community repository (e.g. GitHub). See the Nature Portfolio guidelines for submitting code & software for further information.

Data

Policy information about availability of data

All manuscripts must include a data availability statement. This statement should provide the following information, where applicable:

- Accession codes, unique identifiers, or web links for publicly available datasets
- A description of any restrictions on data availability
- For clinical datasets or third party data, please ensure that the statement adheres to our policy

All data found and analyzed during this study are included in this paper and its supplementary files.

Research involving human participants, their data, or biological material

Policy information about studies with human participants or human data. See also policy information about sex, gender (identity/presentation), and sexual orientation and race, ethnicity and racism.

Reporting on sex and gender	N/A
Reporting on race, ethnicity, or other socially relevant groupings	N/A
Population characteristics	N/A
Recruitment	N/A
Ethics oversight	N/A

Note that full information on the approval of the study protocol must also be provided in the manuscript.

Field-specific reporting

Please select the one below that is the best fit for your research. If you are not sure, read the appropriate sections before making your selection.

☒ Life sciences ☐ Behavioural & social sciences ☐ Ecological, evolutionary & environmental sciences

For a reference copy of the document with all sections, see nature.com/documents/nr-reporting-summary-flat.pdf

Life sciences study design

All studies must disclose on these points even when the disclosure is negative.

Sample size	For the in vivo study, each group consisted of 9 mice, while for the in vitro cell experiment, each group included three to nine.
Data exclusions	N/A
Replication	The experiment was independently repeated a minimum of three times.
Randomization	Mice were randomly allocated to each group at the beginning of the study.
Blinding	The tester did not know the experimental grouping.

Reporting for specific materials, systems and methods

We require information from authors about some types of materials, experimental systems and methods used in many studies. Here, indicate whether each material, system or method listed is relevant to your study. If you are not sure if a list item applies to your research, read the appropriate section before selecting a response.

Materials & experimental systems

n/a	Involved in the study	
☐	☒	Antibodies
☐	☒	Eukaryotic cell lines
☒	☐	Palaeontology and archaeology
☐	☒	Animals and other organisms
☒	☐	Clinical data
☒	☐	Dual use research of concern
☒	☐	Plants

Methods

n/a	Involved in the study	
☒	☐	ChIP-seq
☐	☒	Flow cytometry
☒	☐	MRI-based neuroimaging

Antibodies

Antibodies used	p-p38 (28796-1-AP Polyclonal, Proteintech)<br>p38 (14064-1-AP, Polyclonal, Proteintech<br>p-JNK (80024-1-RR, Polyclonal, Proteintech)<br>JNK2 (PTM-6948, Monoclonal, Proteintech

	IκB (10268-1-AP, Polyclonal, Proteintech?<br>p-ERK (PTM-7155, Monoclonal, PTM Biolabs)<br>ERK (PTM-6324, Monoclonal, PTM Biolabs)<br>P65 (PTM-5254, Monoclonal, PTM Biolabs)<br>Caspase-1 (PTM-6865, Monoclonal, PTM Biolabs)<br>IL-18 (PTM-6235, Monoclonal, PTM Biolabs)<br>ASC (PTM-6894, Monoclonal, PTM Biolabs)<br>IL-1β (A16288, Polyclonal, ABclonal Technology)<br>Cleaved-Caspase1 (D57A2, Monoclonal, Cell Signaling Technology<br>Cleaved-Gasdermin D (E3E3P, Monoclonal, Cell Signaling Technology)<br>NLRP3 (A5652, Polyclonal, ABclonal Technology)<br>β-actin (66009-1-Ig, Monoclonal, Proteintech)<br>Myosin VIIa (M03915, Monoclonal, Boster Bio)
Validation	All antibodies are commercial antibodies purchased from Proteintech, PTM Bio, ABclonal Technology, Cell Signaling Technology and Boster Bio.

Eukaryotic cell lines

Policy information about cell lines and Sex and Gender in Research

Cell line source(s)	House Ear Institute-Organ of Corti 1 (HEI-OC1) were gifted from Institute of Otorhinolaryngology of Jilin University.Mouse squamous cell carcinoma cells (SCC-7) were purchased from Bohui Biotechnology Co.Uppsala 87 Malignant Glioma cells (U-87 MG) were kindly provided by the Institute of Basic Medical Sciences, Jilin University.A549 cells were kindly provided by the Institute of Basic Medical Sciences, Jilin University.
Authentication	Not formally authenticated by STR profiling. However, the characteristic morphology of the HEI-OC1 cells was consistently confirmed by scanning electron microscopy (SEM) analysis throughout our study. Additionally, all cell lines were obtained directly from reputable research institutions and used at low passage numbers to further minimize risks.
Mycoplasma contamination	Not routinely tested. For the cell line HEI-OC1, normal and characteristic morphology was confirmed by scanning electron microscopy (SEM), with no signs of deterioration. All other cell lines were used at low passages from reputable sources and showed no morphological signs of contamination in routine culture.
Commonly misidentified lines<br>(See ICLAC register)	N/A

Animals and other research organisms

Policy information about studies involving animals; ARRIVE guidelines recommended for reporting animal research, and Sex and Gender in Research

Laboratory animals	C57BL/6J male mice (7 weeks old) and Sprague-Dawley (SD) rat pups (4 days old).
Wild animals	The study did not involve wild animals.
Reporting on sex	Findings apply to male mice only. The sex of the 4-day-old rat pups was not determined or considered irrelevant for the cochlear explant culture. Sex-based analysis was not performed.
Field-collected samples	N/A
Ethics oversight	All animal experiments were approved by the Institutional Ethical Committee of Changchun University of Chinese Medicine (Approval No: 2022440).

Note that full information on the approval of the study protocol must also be provided in the manuscript.

Plants

Seed stocks	N/A
Novel plant genotypes	N/A
Authentication	N/A

Flow Cytometry

Plots

Confirm that:

☒ The axis labels state the marker and fluorochrome used (e.g. CD4-FITC).

☒ The axis scales are clearly visible. Include numbers along axes only for bottom left plot of group (a 'group' is an analysis of identical markers).

☒ All plots are contour plots with outliers or pseudocolor plots.

☒ A numerical value for number of cells or percentage (with statistics) is provided.

Methodology

Sample preparation	HEI-OC1 cells were collected, washed with PBS, and suspended in Annexin V binding buffer at a concentration of 2×10^5 cells/ml. Cells were then stained with fluorescein isothiocyanate (FITC)-conjugated Annexin V and Propidium Iodide (PI) for analysis of cell death.
Instrument	BD LSRFortessa™ Flow Cytometer (Make: BD Biosciences; Model: LSRFortessa™)
Software	FlowJo software was used for data analysis.
Cell population abundance	The abundance of viable (Annexin V-/PI-), early apoptotic (Annexin V+/PI-), late apoptotic/dead (Annexin V+/PI+) cell populations was quantified and presented as percentages in the figures. The purity was inherent to the analysis of the entire cell population.
Gating strategy	N/A

☐ Tick this box to confirm that a figure exemplifying the gating strategy is provided in the Supplementary Information.
